# Occurrence and Genomic Characterization of ESBL-, AmpC-, and Carbapenemase-Producing *Escherichia coli* and *Klebsiella pneumoniae* Isolated from Surface Water in Southern Italy, 2023–2024

**DOI:** 10.3390/microorganisms14020508

**Published:** 2026-02-22

**Authors:** Gaia Nobili, Annachiara Cocomazzi, Maria Grazia Basanisi, Annita Maria Damato, Rosa Coppola, Maria Grazia Cariglia, Ilenia Franconieri, Antonella Stallone, Michelina Notarangelo, Tommaso Scirocco, Valeria Bortolaia, Giovanna La Salandra

**Affiliations:** 1 Istituto Zooprofilattico Sperimentale Della Puglia e Della Basilicata, Via Manfredonia 20, 71121 Foggia, Italy; annachiara.cocomazzi@izspb.it (A.C.); annita.damato@izspb.it (A.M.D.); rosa.coppola@izspb.it (R.C.); mariagrazia.cariglia@izspb.it (M.G.C.); giovanna.lasalandra@izspb.it (G.L.S.); 2 Dipartimento Ambientale Provinciale (DAP) Foggia, Agenzia Regionale per la Prevenzione e la Protezione Dell’Ambiente Puglia, Via Giuseppe Rosati 139, 71121 Foggia, Italy; i.franconieri@arpa.puglia.it (I.F.); a.stallone@arpa.puglia.it (A.S.); m.notarangelo@arpa.puglia.it (M.N.); 3 Istituto per le Risorse Biologiche e le Biotecnologie Marine del Consiglio Nazionale Delle Ricerche (IRBIM CNR), Via Pola 4, 71010 Lesina, Italy; tommaso.scirocco@cnr.it; 4 Statens Serum Institut, Artillerivej 5, 2300 Copenhagen, Denmark; vabo@ssi.dk

**Keywords:** antimicrobial resistance, *Escherichia coli*, *Klebsiella pneumoniae*, ESBL, carbapenemases, surface water, WGS

## Abstract

Antimicrobial resistance (AMR) is recognised as a major global public health threat, with the environment increasingly acknowledged as a key reservoir and dissemination pathway for resistant bacteria and resistance genes. In this study, 148 surface water samples were collected between 2023 and 2024 from six rivers and three canals discharging wastewater into two lake waters in Southern Italy to assess the occurrence and genomic features of extended-spectrum β-lactamase (ESBL)-, AmpC-, and carbapenemase-producing *Escherichia coli* and *Klebsiella pneumoniae*. Relevant isolates were obtained using selective culturing, and tested for antimicrobial susceptibility by broth microdilution. Major β-lactam resistance genes were detected by real-time PCR. Whole-genome sequencing (WGS) was performed on presumptive carbapenemase-producing isolates. ESBL- and/or carbapenemase-producing Enterobacterales were detected in 67.6% of samples, yielding a total of 176 non-duplicate isolates. The most prevalent gene was *bla*_CTX-M_, detected in 79.3% of positive isolates (96/121), while carbapenemase genes were detected in 20.6% (25/121) of isolates, mainly *bla*_OXA-48_ and *bla*_VIM_. WGS analysis of carbapenemase PCR-positive isolates revealed occurrence of clinically relevant high-risk clones, such as *K. pneumoniae* ST512/ST307 carrying *bla*_KPC-3_ and *E. coli* ST10 harbouring *bla*_OXA-244_. These findings highlight a potential risk to public health and underscore the importance of integrating environmental compartments into One Health surveillance frameworks for AMR.

## 1. Introduction

Antimicrobial resistance (AMR) has been recognised by the World Health Organization (WHO) as one of the major global health threats, driven by the rise of multidrug-resistant (MDR) bacteria and the progressive reduction of effective therapeutic options [1]. Projections estimate that AMR could cause up to 10 million deaths annually by 2050 [2]. Among Enterobacterales, the predominant mechanism of resistance to β-lactam antibiotics is the production of enzymes capable of hydrolysing and inactivating such antimicrobials [3]. More than 2000 naturally occurring β-lactamase variants have been described to date, including clinically relevant families such as class A penicillinases, extended-Spectrum β-Lactamases (ESBLs; e.g., TEM, SHV, VEB, CTX-M), AmpC cephalosporinases (e.g., CMY, FOX, DHA, ACT, MOX), and carbapenemases belonging either to serine-β-lactamases (e.g., KPC, IMI, SME, GES) or metallo-β-lactamases (e.g., NDM, VIM, IMP) [4]. Environmental detection of antimicrobial-resistant bacteria (ARBs) has received increasing attention due to the potential health risk posed by bacteria associated with humans, livestock or wildlife at the interface with anthropogenic environments [5,6]. Historically, research has focused primarily on clinical, veterinary, and food-producing animal settings. However, over the past decade, the environment has been increasingly recognised as playing a significant role in the development and dissemination of AMR [7,8]. The environment contributes to AMR through two major processes [7]. First, it serves as a vehicle for the dissemination of already resistant bacteria between humans, or between animals and humans. ARBs may be released into the environment through municipal wastewater [9], irrigation with reclaimed water [10], agricultural practices [11], and the application of treated sewage sludge as biosolids [12]. There is evidence that resistant bacteria can subsequently re-enter the human microbiome via environmental exposure [13], for example through ingestion of water contaminated by sewage during recreational activities [14], consumption of fresh produce irrigated with surface water [15,16], or more generally in settings with inadequate sanitation [17]. Second, the environment acts as a reservoir and facilitator for the evolution of AMR. Surface waters can constitute resistance hotspots where antibiotic resistance genes (ARGs) disseminate—favoured by bacteriophages, integrons, and other mobile genetic elements—and new resistant strains can be generated through horizontal gene transfer [18]. Although the presence of β-lactamase-encoding genes in bacteria isolated from surface waters, livestock wastes (including dairy farm effluents), and wastewater treatment plants has been reported in several geographical settings [5,18,19,20], robust and harmonised environmental surveillance data remain limited. In particular, limited data are available for Apulia, a large region in Southern Italy characterised by high population mobility and seasonal tourist influx. The present study aims to assess the occurrence and genomic characteristics of extended-spectrum β-lactamase (ESBL)-, AmpC-, and carbapenemase-producing *Escherichia coli* and *Klebsiella pneumoniae* in surface waters of Apulia with the objective of generating baseline evidence to support environmental antimicrobial resistance surveillance, within a One Health approach.

## 2. Materials and Methods

### 2.1. Sampling Points

**Figure 1:**
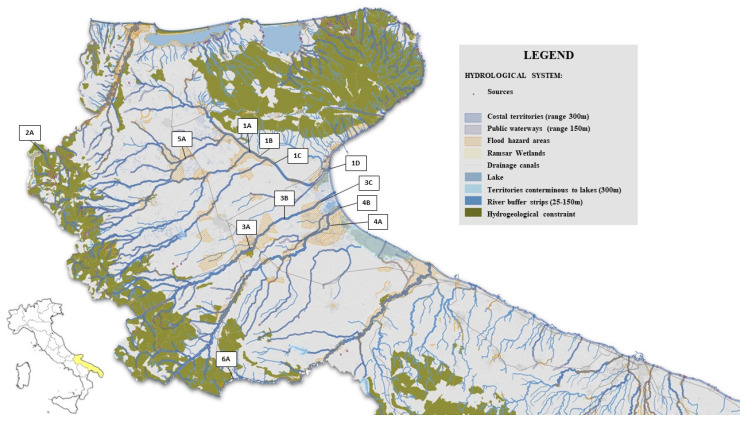
Map of river sampling points.

**Figure 2:**
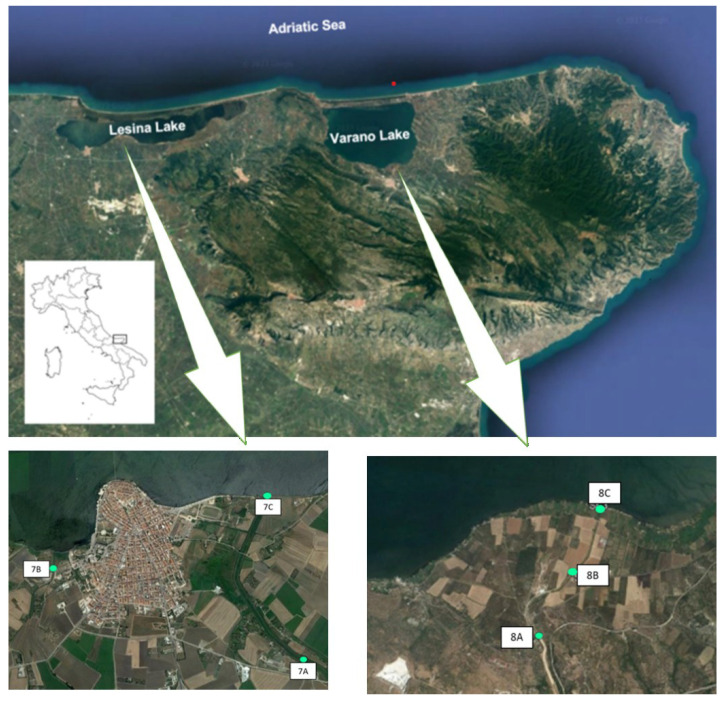
Map of lake sampling points.

Water samples were collected from four rivers, two torrents, and three canals conveying water into two lakes of the Apulia region between 2023 and 2024. The rivers included Candelaro (4 sampling points; 1A, 1B, 1C, 1D), Fortore (1 sampling point; 2A), Cervaro (3 sampling points; 3A, 3B, 3C), and Ofanto (1 sampling point; 6A) rivers (Figure 1 and Table S1); and torrents Carapelle (2 sampling points; 4A, 4B) and Triolo (1 sampling point; 5A). Rivers and torrents were selected for sampling based on the annual mean *E. coli* concentrations recorded in 2021 at each monitoring site, as reported by ARPA Puglia (https://www.arpa.puglia.it/pagina2975_ii-ciclo-sessennale-2016-2021.html, accessed on 14 November 2025). Specifically, sampling sites with the highest average *E. coli* values were prioritised, given the role of *E. coli* as an indicator of faecal contamination in surface waters. In addition, sampling points were also chosen on the basis of their proximity to agricultural land observed using satellite imagery. Sampling stations within the canals were strategically located along channels receiving wastewater discharges from adjacent settlements into the lake systems. Overall, a total of six sampling points were set for this study: three for Lake Lesina (7A, 7B, and 7C) and three for Lake Varano (8A, 8B, and 8C) (Figure 2 and Table S1). For Lesina lake, it was chosen to sample the waters of the Cammarata canal within which wastewater from the settlements of Lesina and Poggio Imperiale are conveyed. Of the three sampling points, two (7A, 7C) were placed along the Cammarata canal: 7A at the outlet of the wastewater treatment plant and 7C at the incile of the Cammarata canal into the lagoon. A third sampling point (7B) was fixed along the La Fara canal, which collects wastewater from an area close to the town of Lesina affected by various agricultural and livestock activities and discharges it into the lagoon (Figure 2). For Varano lake, it was chosen to sample the waters of the San Francesco canal within which wastewater from the town of Cagnano Varano is conveyed. The sampling point 8A is located at the outlet of the wastewater treatment plant, 8B in an area of the canal near which there is a livestock farm, and a third sampling point (8C) at the incile of the San Francesco canal in the lagoon (Figure 2). The analysed canals constitute critical control points for the environmental quality of the lakes, as they play a pivotal role in maintaining the ecological equilibrium of the lagoon system and in safeguarding the safety and long-term sustainability of associated economic activities.

### 2.2. Sampling Procedure

From May 2023 to December 2024, a total of 148 surface water samples were collected from surface water bodies in the Apulia region, including four rivers, two torrents, and three canals conveying water into two lakes (Figure 1, Figure 2, Table S1). For each surface water body, a minimum of ten samples were collected and analysed (Table S1). On each sampling occasion, a volume of 500 mL was collected at each site directly by filling a sterile glass container with surface water. The samples were transferred under refrigerated conditions to the laboratories of the Istituto Zooprofilattico Sperimentale della Puglia e della Basilicata (IZS PB, Foggia, Italy). The samples were stored at 4 °C and analysed within 24 h.

### 2.3. Microbiological Screening, Isolation and Identification of Target Bacteria

The isolation procedure aimed to selectively detect resistant bacteria from the examined samples. For the isolation of target microorganisms, 100 mL of each water sample was filtered (0.45 μm; Sartorius Stedim Biotech GmbH, Goettingen, Germany). To select for ESBL-producing Enterobacterales, the filter was placed in 100 mL of Buffer Peptone Water (BPW) (Thermo Fisher Scientific, Rhone, MI) and incubated at 42 °C for 18–24 h. Subsequently, 100 μL of each dilution (up to 10-3) was plated on CHROMagar ESBL plates (CHROMagar, Paris, France) and incubated at 42 °C for 18–24 h. For the isolation of carbapenemase-producing Enterobacterales (CPE), the filter was placed in 100 mL of Mossel broth (EE Broth) (Scharlab S.L., Sentmenat, Spain) and the enrichment incubated at 42 °C for 18–24 h. Subsequently, 100 μL of each dilution (up to 10-2) was plated on CHROMagar mSuperCARBA plates (CHROMagar, Paris, France) and incubated at 42 °C for 18–24 h. After incubation, up to three presumptive colonies of *E. coli* and *Klebsiella* spp. per sample were subcultured onto Blood Agar plates (Liofilchem, Roseto degli Abruzzi, Italy), incubated at 37 °C for 24 h, and subjected to identification by MALDI-TOF mass spectrometry (Bruker Daltonics, Milan, IT). The isolates were cryopreserved in Microbank (PRO-LAB DIAGNOSTICS, Richmond Hill, ON, Canada) and stored at –80 °C.

### 2.4. Phenotypic Detection of Antimicrobial Resistance

Phenotypic antimicrobial susceptibility was assessed using the broth microdilution method, following EUCAST guidelines (version 15.0; www.eucast.org) [21]. GN4F and EUVSEC2 panels (Thermo Fisher Scientific Diagnostics, Netherlands) were used. Plates were incubated at 37 °C for 24 h, and MICs were read using the Thermo Scientific™ Sensititre™ Vizion™ system. *E. coli* ATCC 25922 and *K. pneumoniae* ATCC 700603 served as quality control strains. Results were interpreted according to epidemiological cut-off values (ECOFFs) provided by the European Committee on Antimicrobial Susceptibility Testing (EUCAST) (www.eucast.org) [21]. Multidrug resistance (MDR) was defined as non-susceptibility to at least one agent in three or more antimicrobial classes, according to Magiorakos et al. [22].

### 2.5. Genotypic Detection of ESBL- and Carbapenemase-Encoding Genes and Characterisation by Whole-Genome Sequencing

Automated bacterial DNA extraction was performed on the Maxwell RSC Extraction System (Promega, USA) using the Maxwell RSC PureFood Pathogen kit (Promega, USA). The quantity (ng/μL) and purities (260/280 and 260/230 ratios) of the extracted DNA were measured spectrophotometrically using the IMPLEN NanoPhotometer N60 (Implen GmbH, München, Germany). DNA from all isolates was used for real-time PCR using the Allplex EnteroDR assay kit for the detection of *bla*_NDM_, *bla*_KPC_, *bla*_OXA-48_, *bla*_VIM_, *bla*_IMP_, *bla*_CTX-M_ genes (Seegene Inc, Seoul, Republic of Korea). DNA from isolates that tested positive in PCR for carbapenemase genes was also characterised by whole-genome sequencing (WGS). Genomic DNA was sequenced using Illumina short-read technology on a NextSeq platform with 2 × 151 bp reads. Short-read assembly was performed with SPAdes v3.15.2. The quality of raw sequencing and assembly data was assessed using the analytical tool BIFROST (https://github.com/ssi-dk/bifrost/). For *K. pneumoniae* strains, species identification, multilocus sequence typing (MLST), resistance gene detection, and plasmid replicon typing were performed using the Pathogenwatch platform (https://pathogen.watch). For *E. coli*, all bioinformatics analyses were performed on the public ARIES Galaxy server [23].

### 2.6. Data Availability and Sequence Deposition

Whole-genome sequencing data generated in this study have been deposited in the NCBI Sequence Read Archive (SRA) under BioProject accession number PRJNA1358062.

## 3. Results

### 3.1. Selective Isolation of Presumptive ESBL- and Carbapenemase-Producing Enterobacterales from Surface Water

Out of 148 water samples analysed, isolates presumptively identified as ESBL- and/or carbapenemase-producing Enterobacterales were recovered from 67.6% (100/148) of the samples. All surface water sites tested positive for presumptive ESBL- and/or carbapenemase-producing *Escherichia coli* and *Klebsiella pneumoniae* on at least one sampling occasion, as indicated by growth on selective media. A total of 176 strains were obtained. When multiple isolates were recovered from the same sampling site across different sampling occasions, one representative strain per sample was selected for downstream analyses. Overall, 57.4% (101/176) of the isolates were *E. coli* and 42.6% (75/176) were *K. pneumoniae.*

### 3.2. Detection of ESBL- and Carbapenemase-Encoding Genes by Multiplex Real-Time PCR

Out of 176 isolates analysed by PCR, 68.8% (121/176) carried at least one of the investigated resistance genes, while 31.2% (55/176) were negative for all targets (Supplementary data, Table S2). Among gene-positive isolates (n = 121), the most prevalent resistance determinant was *bla*_CTX-M_, detected in 79.3% (96/121) of isolates, either as the sole resistance gene or in combination with carbapenemase-encoding genes. Carbapenemase genes were identified in 20.6% (25/121) of isolates, including *bla*_OXA-48_ (3.3%, 4/121), *bla*_KPC_ (1.7%, 2/121), and *bla*_VIM_ (9.1%, 11/121), either alone or in combination with *bla*_CTX-M_ (Table 1).

Among *E. coli* isolates (n = 101), 30.7% (31/101) were PCR-negative for all resistance genes (Table S3). The distribution of resistance genes and gene combinations among *E. coli* and *K. pneumoniae* isolates is summarised in Table 1.

### 3.3. Phenotypic and Genotypic Detection of Antimicrobial Resistance

**Figure 3:**
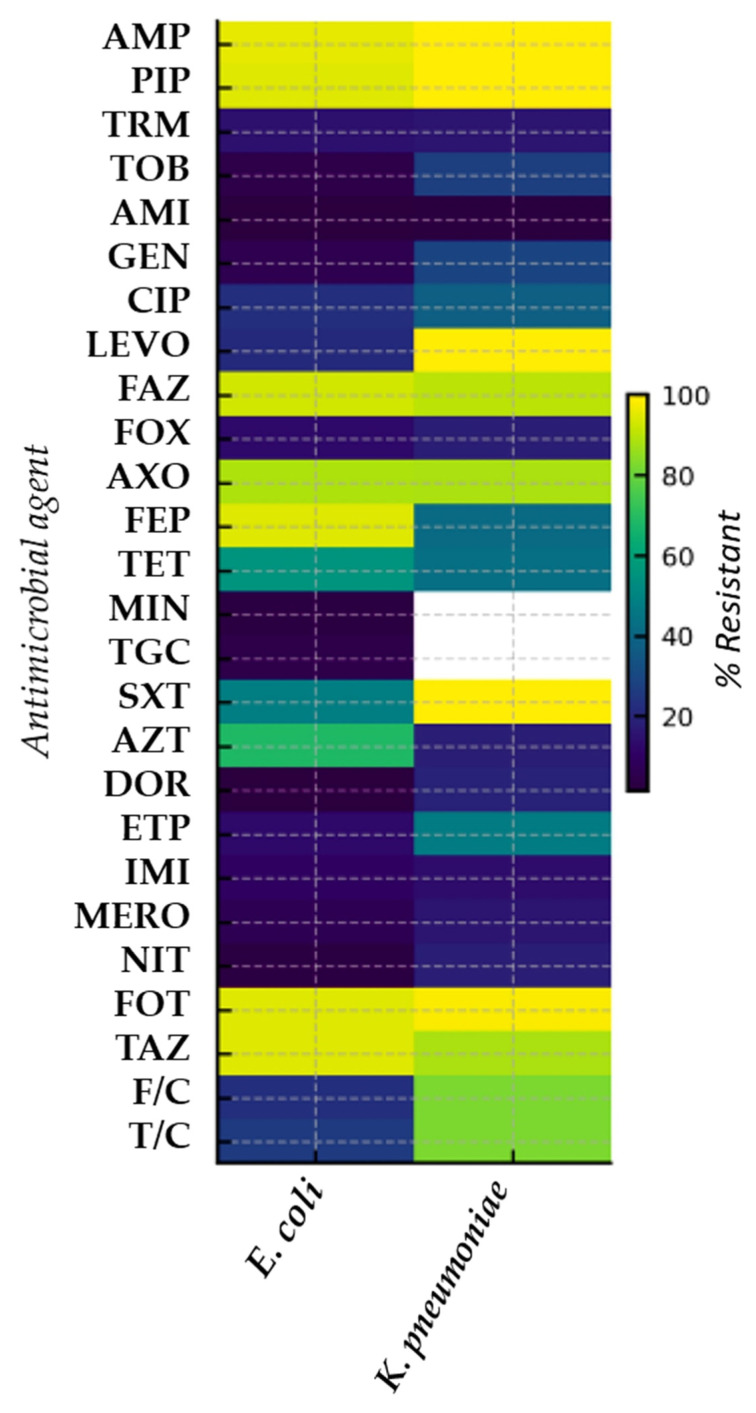
Heatmap of AMR profiles of *E. coli* and *K. pneumoniae* isolated. Antibiotic abbreviations are as follows: AMP, ampicillin; PIP, piperacillin; TRM, temocillin; TOB, tobramycin; AMI, amikacin; GEN, gentamicin; CIP, ciprofloxacin; LEVO, levofloxacin; FAZ, cefazolin; FOX, cefoxitin; AXO, ceftriaxone; FEP, cefepime; TET, tetracycline; MIN, minocycline; TGC, tigecycline; SXT, trimethoprim-sulfamethoxazole; AZT, aztreonam; DOR, doripenem; ETP, ertapenem; IMI, imipenem; MERO, meropenem; NIT, nitrofurantoin; TAZ, ceftazidime; F/C, cefotaxime-clavulanic acid; T/C, ceftazidime-clavulanic acid.

Antimicrobial susceptibility testing was performed on all 176 isolates (Table 2, Figure 3). Among the 31 *E. coli* strains that tested negative for all resistance genes by PCR, 19 isolates were susceptible to all antibiotics tested, 11 showed resistance to up to three antibiotic classes, and only one isolate was multidrug-resistant (Supplementary Data). For *K. pneumoniae*, 24 isolates tested negative in the PCR analysis, and their antimicrobial profiles are reported in the Supplementary Data. Overall, resistance patterns varied between *E. coli* and *K. pneumoniae*, with multidrug resistance observed in both species (Table 3 and Figure 3). Detailed MIC values and breakpoints for each antimicrobial are reported in Supplementary Table S4.

### 3.4. Whole-Genome Sequencing (WGS) of Isolates Positive for Carbapenemase-Encoding Genes by PCR

All (25/121) of Enterobacterales PCR-positive for carbapenemase encoding were subjected to WGS. Detailed isolate-level WGS results are provided in Table 3 for the 25 isolates (56% (14/25) *E. coli* and 44% (11/25) *K. pneumoniae)*, including detected β-lactamase genes (carbapenemases, ESBL/AmpC), additional antimicrobial resistance (AMR) determinants, multilocus sequence types (MLST), and plasmid replicon types. WGS confirmed the presence of carbapenemase-encoding genes in 68% (17/25) of isolates, of which 58.8% (10/17) were *E. coli* and 41.2% (7/17) *K. pneumoniae*. Among *E. coli*, carbapenemase genes were *bla*_OXA-244_ (4/10, 40%), followed by *bla*_VIM-4_ (3/10, 30%), *bla*_OXA-181_ (1/10, 10%), and *bla*_KPC-3_ (2/10, 20%). In *K. pneumoniae*, carbapenemase genes included *bla_KPC-3_* (3/7, 42.85%) and *bla*_VIM_*_-1_* (4/7, 57.14%). ESBL/AmpC genes were detected in 36.0% (9/25) of isolates, including five *E. coli* and four *K. pneumoniae*. The most frequently detected ESBL/AmpC genes were *bla_CTX-M-15_*, *bla_SHV-12_*, *bla_SHV-28_*, *bla_TEM-52B_*, and *bla_DHA-1_*. Multilocus sequence typing (MLST) revealed considerable diversity. High-risk clones such as *K. pneumoniae* ST512 and ST307 carrying *bla*_KPC-3_ and *E. coli* ST10 carrying *bla*_OXA-244_ were identified (Table S4). In addition to β-lactam resistance determinants, multiple acquired resistance genes were detected: 72% of isolates carried aminoglycoside-modifying enzymes (*aac*, *aph*), 64% harboured sulfonamide resistance genes (*sul1* or *sul2*), 60% carried *dfrA* variants (trimethoprim resistance), 44% had macrolide resistance genes (*mph(A)*), and 40% possessed tetracycline resistance genes (tet(A/B/D)). The isolates showed marked genomic diversity, encompassing 12 distinct MLST types (e.g., ST10, ST43, ST307, ST512, ST540, ST746, ST1721), and multiple plasmid incompatibility groups, most frequently IncFIB (56%), IncFIA (48%), IncX1 (28%), IncR (20%), and Col-type plasmids (32%).

## 4. Discussion

Antimicrobial resistance (AMR) in Enterobacterales, including *E. coli* and *K. pneumoniae*, represents a critical global health threat, responsible for significant mortality, morbidity, and economic burden. Environmental reservoirs and human activities play a key role in the persistence and dissemination of AMR, particularly with respect to carbapenem resistance [24,25,26,27]. In this study, we provide evidence of widespread contamination of surface waters in the Apulia region (Southern Italy) with ESBL- and carbapenemase-producing *E. coli* and *K. pneumoniae*. Using selective culture methods, 176 non-duplicate isolates were recovered from 148 samples collected across eight surface water bodies, with ESBL- or carbapenemase-producing *E. coli* or *K. pneumoniae* detected in 68.8% of the analysed samples.

These findings confirm that surface waters can act as important reservoirs and dissemination pathways for AMR, particularly in areas influenced by wastewater discharge and agricultural activities [28,29] and support the integration of environmental surveillance into One Health AMR surveillance frameworks. Marked spatial heterogeneity in *E. coli* concentrations was observed across sampling sites, indicating variable faecal contamination, which may be associated with anthropogenic influences such as wastewater inputs, agricultural runoff, and livestock-related activities. According to previous studies [30,31], *E. coli* was the most frequently isolated species (57.4%), in line with its recognised ubiquity and persistence in aquatic environments [32], where it can act as a reservoir and vector for horizontally transferable resistance genes [33]. The co-occurrence of *K. pneumoniae* (42.6%) further suggests faecal contamination and potential contributions from both human and animal sources [14]. Notably, all sampling sites yielded presumptive ESBL- and carbapenemase-producing strains on at least one sampling occasion, suggesting widespread environmental dissemination of resistant clones. Phenotypic antimicrobial susceptibility testing revealed high occurrence of resistance to multiple antimicrobial classes. The heatmap highlights clear species-specific differences in the distribution of phenotypes across antimicrobial classes. The high levels of resistance observed in both *E. coli* and *K. pneumoniae* to aminopenicillins and extended-spectrum cephalosporins should be interpreted in light of the study design, as isolates were recovered using selective culture media. This targeted isolation approach was intentionally applied to enhance the recovery of resistant Enterobacterales and, by definition, enriches for resistance to the corresponding antimicrobial classes. Consequently, the observed resistance profiles do not reflect the background prevalence of AMR in the sampled environments, but rather the phenotypic characteristics of the selected isolate collection. Nevertheless, the identified resistance patterns are consistent with EU-level surveillance data documenting the widespread occurrence of ESBL-producing Enterobacterales in environmental and food-production settings [21]. The recovery of isolates growing on selective chromogenic media but testing negative for the ESBL- and carbapenemase-encoding genes targeted by PCR can be explained by the intrinsic characteristics of the screening approach. Chromogenic media such as CHROMagar ESBL and CHROMagar mSuperCARBA are designed to maximise sensitivity and may therefore allow the growth of Enterobacterales with reduced β-lactam susceptibility due to intrinsic or low-level resistance mechanisms, even in the absence of classical ESBL or carbapenemase genes. Similar limitations, including reduced specificity and occasional growth of PCR-negative isolates, have been reported previously [34,35]. In addition, phenotypic resistance patterns not explained by the PCR targets may reflect the presence of alternative β-lactam resistance mechanisms not covered by the multiplex assay, such as non-CTX-M ESBLs, chromosomal AmpC overexpression, porin alterations, or efflux mechanisms [35,36]. These findings highlight both the complementary value and limitations of selective culture and targeted molecular assays in environmental AMR surveillance and support the use of broader genomic approaches when detailed characterisation of resistance determinants is required [36]. Comparison between phenotypic antimicrobial resistance and WGS-based predictions showed a high degree of concordance. Among ESBL genes, *bla*_CTX-M_ was the most frequently detected (79.3%), in agreement with its global predominance in both clinical and environmental settings [37,38]. Its similar prevalence in *E. coli* and *K. pneumoniae* suggests sustained selective pressure in the environment, potentially driven by antimicrobial residues and/or untreated wastewater inputs [39]. Carbapenemase genes were detected in 20.6% (25/121) of isolates carrying at least one of the investigated resistance genes, mainly *bla*_VIM_, *bla*_KPC_, and *bla*_OXA-48_-like variants, which are widely disseminated in Europe [40,41,42]. The observed co-occurrence of multiple resistance determinants, including combinations of ESBL- and carbapenemase-encoding genes, indicates the presence of complex resistance profiles within the analysed isolates. While such genetic constellations are commonly associated with mobile genetic elements and horizontal gene transfer in aquatic environments [43], the present findings should be interpreted cautiously, as no specific analyses of plasmids or other mobile genetic elements were performed, and no conclusions can therefore be drawn regarding the underlying mechanisms of gene dissemination.

Whole-genome sequencing of 25 presumptive carbapenemase-producing isolates confirmed carbapenemase genes in 68%, supporting the reliability of PCR screening but also highlighting occasional discrepancies between phenotypic and genotypic methods [36]. Discrepancies between PCR and WGS results likely reflect differences in gene variants, sequencing depth, assembly limitations, or the plasmidic location of resistance determinants. PCR was therefore used as a targeted screening tool, while WGS confirmation was interpreted within its technical constraints. Detected carbapenemase variants included *bla*_KPC-3_, *bla*_VIM-1_, *bla*_VIM-4_, *bla*_OXA-244_, and *bla*_OXA-181_, confirming that surface waters can act as convergence points for multiple resistance genes [44]. WGS also revealed substantial clonal diversity. The detection of high-risk clinical lineages, such as *K. pneumoniae* ST512 and ST307 carrying *bla*_KPC-3_, and *E. coli* ST10 carrying *bla*_OXA-244_, is particularly noteworthy, as these lineages are commonly associated with healthcare-associated outbreaks in Italy and Europe [45,46,47,48]. Their presence in rivers and canals suggests possible bidirectional exchange between clinical environments and natural ecosystems [47,49]. In addition, several sequence types (STs) typically associated with environmental or zoonotic reservoirs were identified, including *E. coli* ST602, ST746, and ST1721 and *K. pneumoniae* ST34, ST45, and ST1537, which have been increasingly reported in surface waters, wastewater effluents, and animal-associated settings [7,50,51,52,53,54]. Freshwater ecosystems receiving inputs from wastewater treatment plants, agricultural effluents, and stormwater runoff are recognised as hotspots of microbial evolution, where multiple selective pressures can promote the emergence and maintenance of resistant strains [17,34]. Plasmid replicon typing showed a predominance of IncF, IncX, and IncN plasmids, which are key vectors for ESBL and carbapenemase dissemination [55,56]. However, due to availability of short-read data only, we could not associate ESBL and carbapenemase genes to plasmid replicons. Overall, the high occurrence of ESBL- and carbapenemase-producing *E. coli* and *K. pneumoniae* that we observed in surface waters of Southern Italy is consistent with—and in some aspects even higher than—what has been reported from other Italian and European regions. In Northern Italy, ESBL-, KPC-, and *mcr*-1.2–producing Enterobacterales have been described in streams, wells, and wastewater treatment plants in the Oltrepò Pavese area, and ESBL-producing *E. coli* and *Klebsiella* spp. carrying *bla*_CTX-M_, including OXA-244-producing ST131 *E. coli* and *bla*_KPC-2_ ST258 *K. pneumoniae*, were detected in surface and groundwaters of the Pavia urban area [57]. **Similar findings** have been reported for Dutch and Nordic surface waters, where multidrug-resistant and ESBL-producing *E. coli* are ubiquitous and frequently belong to internationally disseminated clones [58]. Moreover, KPC-producing Enterobacterales have been repeatedly recovered from the Douro River in Portugal and from Italian rivers such as the Lambro, highlighting rivers as long-term reservoirs of carbapenemase producers [42]. Taken together, these data indicate that the contamination we document in Southern Italy is part of a broader European pattern, in which surface waters impacted by urban and healthcare-related wastewater act as hubs for the persistence and dissemination of high-risk ESBL- and carbapenemase-producing Enterobacterales.

## 5. Conclusions

The findings of this study provide qualitative evidence of the presence and diversity of resistant bacteria and indicate that surface waters in the Apulia region act both as reservoirs of clinically relevant high-risk clones and as dynamic environments supporting the persistence and evolution of environmentally adapted Enterobacterales. This highlights the permeability of ecological boundaries between human, animal, and environmental compartments and reinforces the importance of integrating environmental surveillance into One Health AMR monitoring systems. Future studies integrating quantitative microbial measurements, metagenomic approaches, and exposure assessment would be essential to better characterise the environmental burden of AMR and its implications for human and animal health. Targeted interventions addressing wastewater treatment, agricultural runoff, and environmental contamination are urgently needed to mitigate the environmental spread of carbapenemase-producing Enterobacterales in Italy.

## Figures and Tables

**Table 1:** **Occurrence of ESBL- and carbapenemase-encoding genes in *E. coli* and *K. pneumoniae* isolated on selective agar plates**.

Organism	No. of PCR-Positive Isolates	Resistance Genes in Enterobacterales Isolates						
		*bla*_CTX-M_	CRE * (25/121, 20.6%)					
			*bla*_OXA-48_	*bla*_KPC_	*bla*_VIM_	*bla*_CTX-M_ + *bla*_KPC_	*bla*_CTX-M_ + *bla*_VIM_	*bla*_CTX-M_ + *bla*_OXA-48_
*K. pneumoniae*	51/75 (68%)	40/51 (78.4%)	1/51 (2%)	-	5/51 (9.8%)	3/51 (5.9%)	2/51 (3.9%)	-
*E. coli*	70/101 (69.3%)	56/70 (80%)	3/70 (4.3%)	2/70 (2.9%)	6/70 (8.6%)	-	1/70 (1.4%)	2/70 (2.9%)
Total No.	121/176 (68.8%)	96/121 (79.3%)	4/121 (3.3%)	2/121 (1.7%)	11/121 (9.1%)	3/121 (2.5%)	3/121 (2.5%)	2/121 (1.7%)

* CRE, carbapenem-resistant Enterobacterales.

**Table 2:** **Antimicrobial susceptibility profiles of presumptive ESBL- and carbapenemase-producing Enterobacterales isolates**.

Antibiotic Class/Phenotypic Test *		Molecules	No. of *E. coli* Categorized as Resistant (%)	No. of *K. pneumoniae* Categorized as Resistant (%)
**Beta-lactams**	**Penicillins**	**AMP**	67/70 (95.71)	- **
		**PIP**	67/70 (94.28)	- **
	Penicillin derivatives ***	**TRM**	10/70 (14.28)	8/51 (15.68)
	**First generation cephalosporins**	**FAZ**	65/70 (92.85)	46/51 (90.19)
	**Second-generation cephalosporins**	**FOX**	9/70 (12.85)	9/51 (17.64)
	**Third- and fourth-generation**	**AXO**	62/70 (88.57)	45/51 (88.23)
		**FEP**	66/70 (94.28)	21/51 (41.17)
**Monobactam**		**AZT**	48/70 (68.57)	9/51 (17.64)
**Carbapenems**		**DOR**	1/70 (1.42)	10/51 (19.60)
		**ETP**	9/70 (12.85)	24/51 (47.05)
		**IMI**	7/70 (10.00)	7/51 (13.72)
		**MERO**	7/70 (7.14)	8/51 (15.68)
**Aminoglycosides**		**TOB**	3/70 (4.28)	14/51 (27.45)
		**AMI**	1/70 (1.42)	1/51 (1.96)
		**GEN**	4/70 (5.71)	15/51 (29.41)
**Fluoroquinolones**		**CIP**	16/70 (22.85)	19/51 (37.25)
		**LEVO**	16/70 (21.42)	11/51 (21.56)
**Tetracyclines**		**TET**	39/70 (55.71)	22/51 (43.13)
		**MIN**	2/70 (2.85)	8/51 (15.68)
		**TGC**	3/70 (4.28)	2/51 (3.92)
**Sulfonamides**		**SXT**	34/70 (48.57)	36/51 (70.58)
**Nitrofurans**		**NIT**	2/70 (2.85)	-**
ESBL confirmatory tests *		**FOT**	67/70 (94.28)	50/51 (98.03)
		**TAZ**	67/70 (94.28)	45/51 (88.23)
		**F/C**	16/70 (22.85)	42/51 (82.35)
		**T/C**	18/70 (25.71)	42/51 (82.35)

* The MIC values reported in the table represent the epidemiological cut-off values (ECOFFs) used for categorisation, except for ESBL confirmatory tests, which were interpreted according to EUCAST phenotypic criteria. ** For *K. pneumoniae*, no ECOFFs were applied for ampicillin, piperacillin and nitrofurantoin, as this species is considered intrinsically resistant or not a target organism according to EUCAST. *** Temocillin susceptibility was interpreted as an ESBL screening marker according to EUCAST recommendations.

**Table 3:** **Whole-genome sequencing summary of 25 presumptive carbapenemase-producing Enterobacterales isolated from surface waters**.

ID	Sampling Points	Date	Species	Multiplex PCR Results	Carbapenemase	ESBL/ AmpC	Other AMR Genes	MLST	Plasmid Replicon
IZSPB_EC01	1B	11/23	*E. coli*	*bla_CTX-M_* *bla_OXA-48_*	*bla*_OXA-244_	*bla_CTX-M-15_*	*bla*_TEM-1B_, *qnrS1, aph(6)-Id, aph(3’’)-Ib, sul2, dfrA14, tet(A*)	ST10	IncFIB(K), IncFIB(AP0019)
IZSPB_KP01	1B	06/24	*K. pneumoniae*	*bla_CTX-M,_ bla_OXA-48_*	*bla*_KPC-3_	-	*bla*_TEM-1A_ *, bla*_OXA-9_ *, aadA3, aac(3)-IIa, aadA2, aph(3’)-Ia, aac(6’)-Ib, mph(A), sul1, dfrA12, catA1*	ST512	IncFIB(K) ColRNAI IncQ1, IncFIB(pQil)
IZSPB_EC02	1B	07/24	*E. coli*	*bla_VIM_*	-	*bla_TEM-52B_*	*ant(3’’)-Ia, lnu(F)*	ST602	IncX1 IncI1-I(Alpha)
IZSPB_EC03	1D	02/24	*E. coli*	*bla_OXA-48_*	*bla*_OXA-244_	-	-	ST43	IncFIB(AP0019)
IZSPB_EC04	1D	05/24	*E. coli*	*bla_KPC_*	*bla*_KPC-3_	-	-	ST1139	IncI(Gamma), IncFIB(pQil)
IZSPB_KP02	1D	05/24	*K. pneumoniae*	*bla*_CTX-M_, *bla*_KPC_	*bla*_KPC-3_	*bla_SHV-28_, bla_CTX-M-15_*	*bla*_TEM-1A_ *, bla*_OXA-1_ *, bla*_OXA-9_ *, qnrB1, aac(3)-IIa, aph(6)-Id, aph(3’’)-Ib, sul2, dfrA14, aac(6’)-Ib-cr*	ST307	IncFIB(K), IncN4, IncFIB(pQil)
IZSPB_KP03	2A	02/24	*K. pneumoniae*	*bla_OXA-48_*	-	-	-	ST3442	IncFIB(K)(pCA), RepB
IZSPB_KP04	3B	02/24	*K. pneumoniae*	*bla*_VIM_	*bla*_VIM-1_	*bla_SHV-12_*	*qnrS1, aph(3’)-XV, mph(A), sul1, dfrA14, catB2*	ST1537	IncA
IZSPB_KP05	3B	07/24	*K. pneumoniae*	*bla*_CTX-M_, *bla*_KPC_	*bla*_KPC-3_		*bla*_TEM-1A_ *, bla*_OXA-9_ *, tet(D)*	ST45	IncFIB(K), Col440II, ColRNAI, IncFIB(pQil)
IZSPB_KP06	3B	08/24	*K. pneumoniae*	*bla*_VIM_	-	*bla_DHA-1_*	*bla_OKP-B-2_, qnrB4, sul1, dfrA1*	ST2059	IncFIB(K), IncR, Col440I
IZSPB_KP07	3B	09/24	*K. pneumoniae*	*bla*_VIM_	-	-	-	ST983	negative
IZSPB_EC05	3B	09/24	*E. coli*	*bla*_VIM_	-	-	*bla*_TEM-1B_, aph(3’’)-Ib, tet(B)	ST Novel	IncFIB (AP0019)
IZSPB_EC06	4A	03/24	*E. coli*	*bla*_OXA-48_	*bla*_OXA-181_	-	*bla*_TEM-1B_, *aac(3)-IId, aph(6)-Id, aph(3’)-Ia, aph(3’’)-Ib, aadA5, mph(A), erm(B), sul1, sul2, dfrA17, floR, catA1*	ST542	IncFII, IncX1, Col (BS512), Col156, IncQ1
IZSPB_EC07	5A	02/24	*E. coli*	*bla*_OXA-48_	*bla*_OXA-244_	-	-	ST746	negative
IZSPB_EC08	5A	02/24	*E. coli*	*bla*_VIM_	*bla*_VIM-4_	-	-	ST Novel	IncFIB(K), IncFIA(HI1)
IZSPB_KP08	5A	02/24	*K. pneumoniae*	*bla*_VIM_	*bla*_VIM-1_	-	*aph(3’)-XV, mph(A), sul1, catB2*	ST Novel	repB(R1701), IncFIB(K) (pCA), IncFIB(pKPHS1, Col440I
IZSPB_KP09	5A	03/24	*K. pneumoniae*	*bla*_VIM_	*bla*_VIM-1_	-	*qnrS1, aph(3’)-XV, mph(A), sul1, dfrA14, catB2*	ST34	IncFIB(K), IncN, IncR
IZSPB_EC09	5A	03/24	*E. coli*	*bla*_OXA-48_	*bla*_OXA-244_	*bla_CTX-M-15_*	*bla*_TEM-1B_, *qnrS1, aph(6)-Id, aph(3’’)-Ib, sul2, dfrA14, tet(A)*	ST540	IncFIB(K), IncFIB(AP0019)
IZSPB_KP10	5A	03/24	*K. pneumoniae*	*bla*_CTX-M,_ *bla*_VIM_	-	*bla_CTX-M-15_*	*qnrS1, aph(6)-Id, aadA2, aph(3’)-Ia, aph(3’’)-Ib, mph(A), sul1, sul2, dfrA12, catA2*	ST469	IncFIB(K)(pCA), IncFIB(pKPHS1, RepB
IZSPB_EC10	5A	04/24	*E. coli*	*bla*_CTX-M,_ *bla*_VIM_	*bla*_VIM-4_	-	-	ST1721	negative
IZSPB_KP11	5A	04/24	*K. pneumoniae*	*bla*_VIM_	*bla*_VIM-1_	-	*aph(3’)-XV, sul1, catB2*	ST Novel	IncFIB(K), IncFIA(HI1), IncX1, IncR, Col(pHAD28), Col440I
IZSPB_EC11	5A	06/24	*E. coli*	*bla*_VIM_	*bla*_VIM-4_	-	-	ST1721	IncFIB(pHCM2)
IZSPB_EC12	8A	08/24	*E. coli*	*bla*_VIM_	-	*bla_SHV-12_*	qnrS1, aph(3’’)-Ib, tet(A), floR	ST2144	IncFIA, IncX1, ColpVC, IncFIB(AP0019)
IZSPB_EC13	8B	02/24	*E. coli*	*bla*_KPC_	*bla*_KPC-3_	-	-	ST141	IncN
IZSPB_EC14	8B	08/24	*E. coli*	*bla*_VIM_	-	*bla_SHV-12_*	qnrS1, aph(3’’)-Ib, tet(A), floR	ST2144	IncFII(29), IncFIA, IncX1, ColpVC, IncFIB(AP0019)

## Data Availability

Whole-genome sequencing data generated in this study have been deposited in the NCBI Sequence Read Archive (SRA) under BioProject accession number PRJNA1358062. All other data supporting the findings of this study are available from the corresponding authors upon reasonable request.

## Supplementary Materials

The following supporting information can be downloaded at: https://www.mdpi.com/article/10.3390/microorganisms14020508/s1, Table S1: Antibiotic resistance profiles of *E. coli* isolates negative for all genes tested by PCR.; Table S2: Antibiotic susceptibility testing of *Klebsiella pneumoniae* isolates negative for antimicrobial resistance genes tested by PCR.
