# Supplementary material for: Occurrence and Genomic Characterization of ESBL-, AmpC-, and Carbapenemase-Producing Escherichia coli and Klebsiella pneumoniae Isolated from Surface Water in Southern Italy, 2023–2024
Source: Microorganisms. 2026 Feb 22;14(2):508. doi: 10.3390/microorganisms14020508 (PMC12943021; doi:10.3390/microorganisms14020508)
Supplement: Supplementary file 1 [file microorganisms-14-00508-s001.zip › Table S1.pdf]

Table S1. Sampling points and geographical coordinates.

N.	Sampling points	Surface water bodies of Apulia Region	LAT (degrees, minutes, seconds-milliseconds)	LONG (degrees, minutes, seconds-milliseconds)	Annual average *E. coli* count (CFU/100 mL)[1]	Municipality
**1**	1A	Candelaro River	41°37' 34'' N	15°38' 7'' E	4715,8	San Marco in Lamis
**2**	1B	Candelaro River	41°36' 36'' N	15°40' 4'' E	12374,8	San Marco in Lamis
**3**	1C	Candelaro River	41°35' 58'' N	15°42' 18'' E	2240,0	San Marco in Lamis
**4**	1D	Candelaro River	41°34' 25'' N	15°53' 6'' E	1795,8	Manfredonia
**5**	2A	Fortore River	41°38' 50'' N	15°2' 40'' E	505,6	Casalnuovo Monterotaro
**6**	3A	Cervaro River	41°24' 4'' N	15°39' 8'' E	679,0	Foggia
**7**	3B	Cervaro River	41°25' 37'' N	15°40' 4'' E	6228,8	Foggia
**8**	3C	Cervaro River	41°31' 17'' N	15°53' 55'' E	516,7	Manfredonia
**9**	4A	Carapelle Torrent	41°23' 51'' N	15°48' 51'' E	245,1	Cerignola
**10**	4B	Carapelle Torrent	41° 29' 26'' N	15°55' 14'' E	2420,8	Zapponeta
**11**	5A	Triolo Torrent	41° 38' 51'' N	15°32' 44'' E	7749,2	Rignano Garganico
**12**	6A	Ofanto River	41° 05' 29'' N	15° 34' 20'' E	1434,3	Rocchetta S. Antonio
**13**	7A	Cammarata canal (Lesina lake)	41° 51' 34'' N	15°21' 27'' E	data not available	Lesina
**14**	7B	La Fara canal (Lesina lake)	41° 51' 48'' N	15° 21' 24'' E	data not available	Lesina
**15**	7C	Cammarata canal (Lesina lake)	41° 51' 54'' N	15°21'19'' E	data not available	Lesina
**16**	8A	San Francesco canal (Varano lake)	41° 50 '17'' N	15°46' 32'' E	data not available	Varano
**17**	8B	San Francesco canal (Varano lake)	41°50'34''N	15°45'07'' E	data not available	Varano
**18**	8C	San Francesco canal (Varano lake)	41°50'37'' N	15°46'25''E	data not available	Varano

[1].A limit of < 5,000 CFU/100 mL is recommended.
