# Supplementary material for: Occurrence and Genomic Characterization of ESBL-, AmpC-, and Carbapenemase-Producing Escherichia coli and Klebsiella pneumoniae Isolated from Surface Water in Southern Italy, 2023–2024
Source: Microorganisms. 2026 Feb 22;14(2):508. doi: 10.3390/microorganisms14020508 (PMC12943021; doi:10.3390/microorganisms14020508)
Supplement: Supplementary file 1 [file microorganisms-14-00508-s001.zip › Table S2 and S3.pdf]

Supplementary data

Table S2. Antibiotic resistance profiles of *E. coli* isolates negative for all genes tested by PCR.

N.	DATE	ID	FROM	Isolated from	Penicillins		Amynoglicosides			Fluoroquinolones		Cephems (Cephalosporins I)	Cephems Cephalosporins II)	Cephems (Cephalosporins III)		Tetracyclines		Sulfonamides		Monobactam	Carbapenems				Nitrofurans	Penicillins	Combination test			
					AMP	PIP	TOB	AMI	GEN	CIP	LEVO	FAZ	FOX	AXO	FEP	TET	MIN	TGC	SXT	AZT	DOR	ETP	IMI	MEM	NIT	TRM	FOT	F/C	TAZ	T/C
1	08/2023	EC2	4A	ESBL CHR																										
2	03/2024	EC38	1D	mSuperCarba CHR																										
3	05/2024	EC45	7A	mSuperCarba CHR																										
4	06/2024	EC51	1D	ESBL CHR																										
5	06/2024	EC54	7C	ESBL CHR																										
6	07/2024	EC59	6A	ESBL CHR																										
7	08/08/2024	EC64	7B	mSuperCarba CHR																										
8	08/2024	EC66	1B	ESBL CHR																										
9	08/2024	EC68	6A	mSuperCarba CHR																										
10	09/2024	EC70	1A	ESBL CHR																										
11	09/2024	EC71	1B	ESBL CHR																										
12	09/2024	EC74	4A	ESBL CHR																										
13	09/2024	EC76	2A	ESBL CHR																										
14	10/2024	EC77	5A	ESBL CHR																										
15	10/2024	EC78	4A	ESBL CHR																										
16	10/2024	EC79	1D	ESBL CHR																										
17	10/2024	EC80	4B	mSuperCarba CHR																										
18	10/2024	EC81	6A	ESBL CHR																										

19	10/2024	EC82	1C	ESBL CHR																										
20	10/2024	EC83	2A	ESBL CHR																										
21	10/2024	EC84	1B	ESBL CHR																										
22	11/2024	EC86	1B	ESBL CHR																										
23	11/2024	EC87	1C	ESBL CHR																										
24	11/2024	EC89	6A	ESBL CHR																										
25	11/2024	EC90	5A	ESBL CHR																										
26	12/2024	EC91	1D	mSuperCarba CHR																										
27	12/2024	EC92	4A	mSuperCarba CHR																										
28	12/2024	EC95	8C	mSuperCarba CHR																										
29	12/2024	EC96	7A	mSuperCarba CHR																										
30	12/2024	EC97	7B	mSuperCarba CHR																										
31	12/2024	EC98	7C	mSuperCarba CHR																										

Table S3. Antibiotic susceptibility testing of *Klebsiella pneumoniae* isolates negative for antimicrobial resistance genes tested by PCR.

N.	DATE	ID	FROM	Isolated from	Penicillins		Amynoglicosides			Fluoroquinolones		Cephems (Cephalosporins I)	Cephems (Cephalosporins II)	Cephems (Cephalosporins III)		Tetracyclines		Sulfonamides		Monobactam	Carbapenems				Nitrofurans	Penicillins	Combination test			
					AMP	PIP	TOB	AMI	GEN	CIP	LEVO	FAZ	FOX	AXO	FEP	TET	MIN	TGC	SXT	AZT	DOR	ETP	IMI	MEM	NIT	TRM	FOT	F/C	TAZ	T/C
1	19/09/2023	KP1	7C	ESBL CHR																										
2	22/09/2023	KP3	5A	ESBL CHR																										
3	29/11/2023	KP13	1B	ESBL CHR																										
4	04/03/2024	KP24	6A	ESBL CHR																										
5	13/05/2024	KP33	3B	ESBL CHR																										
6	29/07/2024	KP46	5A	mSuperCarba CHR																										

7	30/08/2024	KP50	1B	ESBL CHR																										
8	06/09/2024	KP51	1D	mSuperCarba CHR																										
9	26/09/2024	KP52	1A	ESBL CHR																										
10	26/09/2024	KP53	1B	ESBL CHR																										
11	26/09/2024	KP56	4A	mSuperCarba CHR																										
12	30/09/2024	KP57	6A	mSuperCarba CHR																										
13	07/10/2024	KP59	5A	mSuperCarba CHR																										
14	07/10/2024	KP60	4A	ESBL CHR																										
15	14/10/2024	KP61	1D	ESBL CHR																										
16	14/10/2024	KP62	6A	ESBL CHR																										
17	18/10/2024	KP63	1C	ESBL CHR																										
18	28/10/2024	KP64	1B	ESBL CHR																										
19	25/11/2024	KP66	1B	mSuperCarba CHR																										
20	25/11/2024	KP67	1C	mSuperCarba CHR																										
21	25/11/2024	KP68	1D	mSuperCarba CHR																										
22	29/11/2024	KP69	5A	mSuperCarba CHR																										
23	09/12/2024	KP70	4A	mSuperCarba CHR																										
24	10/12/2024	KP73	7A	mSuperCarba CHR																										
