# Supplementary material for: Occurrence and Genomic Characterization of ESBL-, AmpC-, and Carbapenemase-Producing Escherichia coli and Klebsiella pneumoniae Isolated from Surface Water in Southern Italy, 2023–2024
Source: Microorganisms. 2026 Feb 22;14(2):508. doi: 10.3390/microorganisms14020508 (PMC12943021; doi:10.3390/microorganisms14020508)
Supplement: Supplementary file 1 [file microorganisms-14-00508-s001.zip › Table S4.pdf]

Table S4. Detailed MIC data of *E. coli* and *K. pneumoniae*

Antimicrobial class/confirmatory test[a]	Molecules[a]		MIC* (mg/L) *E. coli*	MIC* (mg/L) *K. pneumoniae*
Beta- lactams	Penicillins	AMP	>8	**
		PIP	>8	**
	Penicillin/beta-lactamase inhibitor***	TRM	>16	>8
	First generation cephalosporins	FAZ	>4	>4
	Second-generation cephalosporins	FOX	>1	>8
	Third- and fourth-generation	AXO	>2	>2
		FEP	>0.25	>0.125
	Monobactam	AZT	>0.25	>4
	Carbapenems	DOR	>0.06	>0.125
		ETP	>0.06	>0.03
		IMI	>0.5	>1
		MERO	>0.06	>0.125
Aminoglycosides		TOB	>4	>2
		AMI	>8	>8
		GEN	>2	>2
Fluoroquinolones		CIP	>0.06	>0.125
		LEVO	>0.125	>0.25
Tetracyclines		TET	>8	>8
		MIN	>4	>8
		TGC	>0.5	>2
Sulfonamides		SXT	>4	>4
Nitrofurans		NIT	>64	**
ESBL confirmatory tests[a]		FOT	>0.25	>0.25
		TAZ	>1	>1
		F/C	>0.25	>0.25
		T/C	>1	>0.5

* The MIC values reported in the table represent the epidemiological cut-off values (ECOFFs) used for categorisation, except for ESBL confirmatory tests, which were interpreted according to EUCAST phenotypic criteria. **For *K. pneumoniae*, no ECOFFs were applied for ampicillin, piperacillin and nitrofurantoin, as this species is considered intrinsically resistant or not a target organism according to EUCAST. *** Temocillin susceptibility was interpreted as an ESBL screening marker according to EUCAST recommendations.
